# Perioperative Rehabilitation in Collaboration with the Department of Occupational Medicine for Patients with Cholangiocarcinoma: A Case Report

**DOI:** 10.1298/ptr.E10275

**Published:** 2024-04-23

**Authors:** Hiroaki TERAMATSU, Akiko HACHISUKA, Masako NAGATA, Shiro KOHI, Manabu HAMADA, Satoshi KUHARA, Akio TAKEMOTO, Hideaki ITOH, Satoru SAEKI

**Affiliations:** ^1^Department of Rehabilitation, University Hospital of Occupational and Environmental Health, Japan; ^2^Department of Rehabilitation Medicine, University of Occupational and Environmental Health, Japan; ^3^Department of Occupational Medicine, University of Occupational and Environmental Health, Japan; ^4^Department of Surgery 1, University of Occupational and Environmental Health, Kitakyushu, Japan

**Keywords:** Cholangiocarcinoma, Perioperative rehabilitation, Department of Occupational Medicine, Return to work

## Abstract

Introduction: Although the number of cancer survivors has increased, the role of physical therapy in return to work (RTW) for employed patients with cancer remains unclear. Case presentation: The patient is a 50-year-old man diagnosed with cholangiocarcinoma who worked as a liquefied petroleum gas station filler. He started perioperative rehabilitation and underwent pancreaticoduodenectomy for cholangiocarcinoma. He developed a postoperative pancreatic fistula, which improved with conservative treatment over 40 days. Although he achieved independence regarding day-to-day activities, his physical condition and workability worsened, as his skeletal muscle index decreased from 8.7 to 7.7, 6-min walk distance from 518 to 460 m, and work ability index (WAI) from 37 to 20 points. His physical therapist was concerned about his RTW and recommended that he receive RTW support from the Department of Occupational Medicine (DOM). The DOM employed a team approach for the RTW strategy, and the primary physician, occupational physician, and company collaborated to support the patient. After the outpatient treatment protocol and RTW support plans were formulated, the patient was discharged. The physical therapist reported declining physical performance and WAI at the DOM’s multidisciplinary conference. After consulting with multiple professionals, the team recommended work resumption in stages: part-time for three months and full-time for four months after surgery while undergoing oral adjuvant chemotherapy. The WAI improved to 35 points after RTW. Conclusion: This case report suggests that physical therapists are vital in providing continuous patient support, from perioperative rehabilitation to DOM intervention, to build physical strength for return to work.

## Introduction

According to the World Health Organization (WHO), one in five persons will develop cancer in their lifetime^1)^. Early cancer detection and development of treatment techniques have improved life expectancy and increased the number of cancer survivors, with approximately half of cancer survivors estimated to be employed^2)^. For cancer survivors, a worsening employment environment, including layoffs, reduces their quality of life and leads to socio-economic losses^3^^,^^4)^. In addition, Japan is an aging society with a declining birthrate, and by 2040, the proportion of the working-age population (15–64 years old) is expected to decrease further^5)^. As a countermeasure to the anticipated increase in social security costs, the Japanese government has implemented certain measures to help cancer survivors return to work (RTW) as one of its primary policies^6)^. Therefore, employment support is crucial for cancer survivors.

Gastrointestinal cancer is highly prevalent, and pancreaticoduodenectomy is one of the most challenging surgical procedures for cholangiocarcinoma. Recent advances in surgical techniques and perioperative management have reduced the surgery-related mortality rate of pancreaticoduodenectomy; however, the incidence of postoperative complications remains extremely high^7)^. Postoperative complications are a disincentive to resume work^8)^.

In a systematic review, perioperative respiratory physical therapy was reported to be effective in preventing postoperative complications of thoracoabdominal surgery and is widely practiced in Japan^9)^. However, the Japanese medical system does not approve outpatient rehabilitation for cancer patients, making it difficult for the patients to obtain rehabilitation support, including RTW assistance, after discharge.

In Japan, from 2018, it became possible to calculate medical payments when medical institutions provide employment support to cancer patients^10)^. In 2018, the Department of Occupational Medicine (DOM) was established at our hospital to provide the patients with health and employment support^11)^. The DOM comprises a multidisciplinary team (physicians, rehabilitation staff, nurses, medical social workers, etc.) of qualified health and employment support coordinators. Nevertheless, the role of physical therapy in RTW for employed patients with cancer remains unclear.

Herein, we aim to clarify the role of physical therapy in the process of RTW for cancer patients based on our experience with a case of perioperative rehabilitation in collaboration with the DOM in a patient with cholangiocarcinoma.

## Case Presentation

### Patient

The patient is a 50-year-old man (height: 172 cm, weight: 74.0 kg, body mass index: 25.0 kg/m^2^). Two months ago, he had no symptoms other than jaundice and was diagnosed with distal cholangiocarcinoma at another hospital. He was subsequently admitted to our hospital for surgery. Laboratory tests showed increased aspartate aminotransferase (AST), alanine aminotransferase (ALT), and total bilirubin levels (AST: 34 IU/L, ALT: 63 IU/L and total bilirubin 2.4 mg/dL). Computed tomography (CT) revealed dilatation of the biliary tree and thickening of the common bile duct wall but no findings suggestive of metastasis. Endoscopic retrograde cholangiography (ERC) showed stenosis of the distal portion of the common bile duct. A biopsy of the distal bile duct showed adenocarcinoma cells. Based on these results, we diagnosed the patient with distal cholangiocarcinoma with clinical stage IIB (cT3N0M0) using the 7th edition of the Japanese classification of biliary tract cancers. The patient worked as a liquefied petroleum gas (LPG) station filler for 30 years. The patient had to return to work following surgery. The company has 480 employees, including a part-time occupational physician, occupational health nurse, and health supervisor, with eight employees at their branch office. Two work shifts (8:00–17:00 hours and 11:00–20:00 hours) are used. His work involved holding hoses and connectors and filling LPG vehicles with gas, which has an estimated metabolic equivalent (MET) value of 3.0^12)^. During busy periods, gas-filling work sometimes continued for over two hours without rest. He commuted for 30 min on foot and 15 min on the subway. As he was performing his daily life and work with no difficulties, he did not feel the need for DOM support and declined assistance during the preoperative interview.

### Preoperative physical therapy intervention

Upon admission, the patient was prescribed preoperative physical therapy to prepare his physical condition for surgery. Preoperative training, comprising five 60-min sessions per week for two weeks, was conducted in the rehabilitation room. The training consisted of breathing exercises, strength training, endurance training using a bicycle ergometer, and self-training guidance (Table 1). For preoperative physical performance assessment, the skeletal muscle index, knee extension strength, grip strength, 6-min walk distance, and Barthel index were measured. The skeletal muscle index was calculated using a body composition meter (InBody S-10; InBody Japan, Tokyo, Japan). Knee extension and grip strength were used to assess muscle strength. A handheld dynamometer (μTas MT-1; ANIMA, Tokyo, Japan) was used to measure the knee extension muscle strength. A Jamer-type hydraulic grip dynamometer (SH5001; SAKAI Medical, Tokyo, Japan) was used to measure grip strength. Details of the physical performance assessment were described in previous reports^13^^,^^14)^. The results of the physical performance assessments are presented in Table 2. The patient’s preoperative physical performance was favorable. The work ability index (WAI) was assessed to determine his work ability before surgery, which was rated as “good.” (Table 3).

**Table 1.** Physical therapy program

Preoperative periods (two weeks)	
Five sessions/week, 60-min/session, in gymnasiums	
Breathing exercise	Coughing, huffing, abdominal breathing exercises
Strength training	Squats, calf raises (100 repetitions)
Endurance training	Moderate intensity cycle ergometer (20–30 min)
Self-training guidance	Necessity and methods of early postoperative weaning
	Physical activity (at least 10,000 steps per day)
Postoperative periods (POD 1–4)	
Five sessions/week, 20-min/session, early mobilization in the ward	
POD 1	Wheelchair transfers
POD 2	50 m walks
POD 3	100 m walks
POD 4	200 m walks
Postoperative complication period (POD 5–14)	
Five sessions/week, 20-min/session, at the bedside, under sustained drainage	
Conditioning	Stretching, massage
Mobilization	Toilet walk—200 m walk, depending on physical condition
Sustained drainage periods (POD 15–42)	
Five sessions/week, 40-min/session, in gymnasiums, under sustained drainage	
Resistance training	Squats, calf raises (30–100 repetitions)
Endurance training	Light-to-moderate intensity interval cycle ergometer (10–20 min) 200–500 m walks
Self-training guidance	Physical activity (at least 5,000 steps per day)
Drain removal—discharge period (POD 43)	
Self-training guidance	Squats, calf raises (100 repetitions)
	Physical activity (at least 10,000 steps per day)
	Maintain the activities of daily life and prepare to resume work

POD, postoperative day

**Table 2.** Changes in physical performance parameters during hospitalization

	Preoperative	Discharge
BW (kg)	74.0	68.7
Skeletal muscle index (kg/m^2^)	8.7	7.7
Knee extension strength (kgf/BW)	0.51/ 0.49	0.60/0.47
Grip strength (kg) (Rt./Lt.)	32/32	34/36
6-min walk distance (m) (Rt./Lt.)	518	460
Barthel index (pts)	100	100

BW, body weight; Rt., right; Lt., left; pts, points

**Table 3.** Change in work ability index from hospitalization to return to work

S. No.	WAI dimensions	Preoperative	Discharge	After RTW
1	Subjective estimation of present work ability compared with lifetime best	8	1	7
2	Subjective work ability in relation to both the physical and mental demands of work	7	2	6
3	Number of diagnosed diseases	4	4	3
4	Subjective estimation of work impairment due to diseases	6	4	7
5	Sickness absenteeism during the past year	2	2	2
6	Own prognosis of work ability after 2 years	7	4	7
7	Enjoying daily tasks; active and alert; full hope for the future	3	3	3
	WAI summary score	37	20	35

WAI, work ability index; RTW, return to work

### Postoperative course

Fourteen days after admission, the patient underwent planned pancreaticoduodenectomy with portal vein resection for cholangiocarcinoma. Postoperative physical therapy was initiated on the day after surgery. Early mobilization progressed in stages, starting with wheelchair transfers, then 50 m, 100 m, and 200 m walks in the ward (Table 1).

However, on postoperative day (POD) 5, the patient developed a pancreatic fistula with fever, and open drainage was performed. The patient was forced to rest, and postoperative physical therapy was performed at the bedside according to his physical condition. On POD 15, the fever improved, and he started gymnasium rehabilitation with limb strength and endurance training in a light-load interval format under sustained drainage. However, residual fatigue rendered active exercise difficult (Table 1).

As a result, his skeletal mass index decreased from 8.7 to 7.7 kg/m^2^, the 6-min walking distance declined from 518 to 460 m (predicted reduction of METs from 3.89 to 3.55)^15)^, and his WAI summary score was reduced from 37 to 20 points) before discharge (Table 2). The patient began to express intense anxiety due to fatigue and weakness during physical therapy. The patient said, “I feel that my physical strength is weakening. I am so exhausted even in my daily life and worried about returning to work.” His physical therapist reported that his physical condition was sufficient for day-to-day activities but not for working. Therefore, the physical therapist recommended a self-training program at the time of discharge, including 1) muscle strengthening training with squats and calf raises with 100 repetitions, 2) physical activity (at least 10000 steps per day^16)^), and 3) maintaining the activities of daily life and preparing to resume work (Table 1). Furthermore, the physical therapist recommended a DOM intervention for the patient. The patient accepted the DOM intervention, which was initiated.

### DOM intervention

The patient was discharged on POD 43. Since outpatient rehabilitation for cancer patients is not approved by the medical system in Japan; hence, at discharge, the patient was instructed to perform the following at home: 1) continue the resistance training that was performed during hospitalization, 2) carry a pedometer and aim to complete at least 10000 steps per day^16)^, and 3) maintain the activities of daily life and prepare to resume work.

**Fig. 1.**
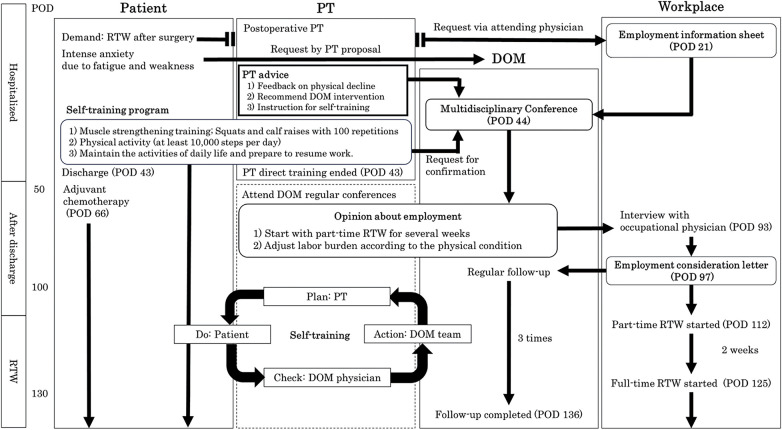
Flow diagram from discharge to RTW RTW, return to work; DOM, department of occupational medicine; POD, postoperative day; PT, physical therapy; ADL, activity of daily living

The flow diagram from discharge to RTW is shown in Figure 1. Before surgery, the patient requested that his attending physician issue an opinion letter to facilitate the RTW. Therefore, the attending physician requested employment information from the workplace, and on POD 21, an employment information sheet was provided by the employer. The employment information sheet stated that the RTW requirements were the ability to 1) commute to work independently using public transportation and 2) communicate and work outdoors using the hands and feet. On POD 44, the DOM convened a multidisciplinary conference. At the meeting, the physical therapist reported that the patient’s physical performance and work ability decreased after surgery but that he could resume work physically, with a reduced workload, given that the estimated METs value for the job was 3.0. In addition, the physical therapist requested that DOM team members check the implementation of the patient’s self-training program during regular visits to the DOM. At the regular DOM clinic, the patient said “I have been able to continue the self-training program without any problems.” while showing his pedometer, and the DOM team shared the training progress at regular DOM meetings.

The attending physician reported that adjuvant chemotherapy was scheduled. After consulting with multiple professionals, it was determined that the patient could perform the work from a medical perspective. In addition, the DOM recommended the following reasonable accommodations for RTW: 1) starting with reduced working hours, mainly in the morning, for several weeks after RTW, and 2) adjusting labor burden according to the physical condition. Our decision is reflected in a letter of opinion regarding employment. The patient said, “I am grateful for DOM’s continuous support. I am anxious about the side effects now that chemotherapy is starting, but I will do my best in the self-training program to get back to work.”

### Progress after DOM intervention

On POD 66, oral fluoropyrimidines (S-1) was initiated as adjuvant chemotherapy. After S-1 administration, the patient did not experience any noticeable side effects. Based on the letter of opinion regarding employment, the occupational physician interviewed the patient on POD 93. During the interview, the following employment considerations were determined: 1) to start with short morning-only shifts and transition to full RTW over three weeks and 2) to take a break indoors every hour during hot weather. On POD 97, an employment consideration letter was submitted to the workplace. Three regular medical examinations were conducted by the DOM until the patient resumed work, and lifestyle and exercise habits were reviewed in preparation for his RTW. The patient said, “My return to work is getting closer, but I am vaguely worried about whether I will be able to do it. Anyway, I will not know until I try so that I will do my best.”

On POD 112, the patient resumed the morning shift, which was expanded to 6 h, one week later. One week after starting the reduced working hours (POD 125), the patient completely resumed regular work without overtime. After confirming that there would be no issues with continuing to work after his RTW, the patient completed the DOM assessment on POD 136. All WAI items and summary scores that decreased after surgery improved after RTW (Table 3). The patient said, “Thanks to DOM's outreach to my workplace and the occupational physician, I was able to return to work with peace of mind. Fortunately, my boss and colleagues also supported me a lot.”

## Discussion

In this case, we performed perioperative rehabilitation in collaboration with the DOM in a patient with cholangiocarcinoma, and two crucial clinical findings were obtained. First, perioperative rehabilitation in collaboration with the DOM may be effective in facilitating a smooth RTW in patients with reduced exercise capacity and work ability following surgery. Second, physical therapists should actively promote health and employment support.

In this patient with cancer, who experienced reduced exercise capacity and work ability after surgery, incorporating an exercise component into the RTW support may have facilitated the seamless transition of exercise instruction from inpatient to outpatient treatment and enabled a smooth RTW. Pancreaticoduodenectomy has a high risk of postoperative complications, particularly pancreatic fistulas, which require long-term drainage^2)^. A previous study reported postoperative complications as factors preventing RTW in cancer patients^8)^. This patient also required prolonged drainage for 41 days and showed decreased weight, skeletal muscle mass, exercise capacity, and work ability. In addition, because the medical system does not approve outpatient rehabilitation for patients with cancer, physical therapy ended after the patient was provided with the instructions for self-training exercises. It has been reported that aerobic and resistance training-driven exercise interventions for working patients with cancer provide a higher RTW effect than usual care, thereby increasing awareness regarding the importance of rehabilitation interventions^17)^. However, unlike rehabilitation programs for stroke and other diseases, outpatient rehabilitation after abdominal surgery is not well-established in clinical settings^18)^. Comprehensive physical, psychological, and occupational interventions are reportedly effective in aiding RTW in breast cancer survivors^19)^.

The physical therapist plays a crucial role in providing perioperative rehabilitation and recommending DOM intervention to the patients, enabling timely DOM intervention. There are few reports on the involvement of physical therapists in the RTW of cancer patients, and most involve patients with breast cancer^20^^,^^21)^. Our previous study also revealed that preoperative physical performance is related to postoperative RTW in patients with lung cancer^11)^. When providing DOM support, a patient request is required for the intervention of the DOM^6)^. However, patients rarely recognize this need, even when they are in a situation that requires professional intervention. In our previous research on perioperative working patients with lung cancer, only 3 of 59 patients (5%) were offered DOM intervention^11)^. It is crucial for physical therapists, who spend considerable time in contact with patients during physical therapy, to determine the need for intervention and promote health and employment support by specialists based on the patient’s challenges and physical performance, and to actively recommend DOM intervention to patients.

A systematic review has reported that perioperative rehabilitation during abdominal surgery is effective in preventing pulmonary complications^22)^. It has been reported that patients with sarcopenia before pancreaticoduodenectomy are at risk of developing postoperative pancreatic fistula and have a poor prognosis^23^^,^^24)^. Although this patient did not have obvious sarcopenia preoperatively, he was administered aggressive physical therapy preoperatively, considering the high risk of postoperative complications due to disease characteristics. As a result, although the patient did not develop any postoperative pulmonary complications, the fever, and prolonged drainage due to a pancreatic fistula resulted in a decline in physical performance at the time of discharge. Preoperative rehabilitation for patients undergoing pancreaticoduodenectomy has been reported to significantly reduce the incidence of postoperative pancreatic fistula^25)^. Comprehensive perioperative rehabilitation is needed to prevent the development of postoperative pancreatic fistulas.

In addition to the decline in physical function after surgery, our patient was scheduled to continue chemotherapy for an extended period after his RTW, and there were concerns about physical effects such as fatigue and decreased physical performance due to side effects associated with chemotherapy. Although Japan’s medical system does not approve outpatient rehabilitation for cancer patients, the DOM team with the physical therapist surmounted this challenge by checking the self-training progress when the patient visited the DOM clinic every fortnight. During outpatient visits after discharge, the physical therapist attended DOM meetings regularly and provided continuous support by sharing the patient’s physical function and self-training methods from the professional perspective of physical therapy. Based on these meetings, the physician in charge of the regular DOM follow-up clinic took over exercise instruction. In this case, the direct and indirect contributions of the physical therapist enabled the patient to build physical strength for return to work. Previous studies reported that work did not necessarily lead to improved health and physical performance among cancer survivors and that continuous support is required even during work^26)^. In the future, it may be necessary to consider including regular physical performance assessments and rehabilitation in the employment support protocol after RTW.

In Japan, very few facilities like our hospital possess a DOM that specializes in promoting health and employment support. If there is no intervention from the DOM, health and employment support is a personal matter between the attending physician and the workplace. In particular, small businesses that do not have occupational physicians often struggle to decide whether to resume work, provide support methods, and cooperate with medical institutions^6)^. The Japan Organization of Occupational Health and Safety is taking the initiative to train coordinators to promote health and employment support. In the future, many physical therapists will be required to champion the promotion of health and employment support and adopt the role of Coordinator of the Promotion of Health and Employment Support.

## Conclusion

There are few reports on physical therapy in the promotion of health and employment support, and there are no established physical therapy programs. In particular, the Japanese medical system does not approve outpatient rehabilitation for cancer patients, making it difficult for them to obtain rehabilitation support, including RTW assistance, after discharge. This case report suggests that physical therapists play a crucial role in providing continuous support for patients, from perioperative rehabilitation to DOM intervention to build physical strength for return to work.

In collaboration with the DOM, perioperative rehabilitation may contribute to a smooth RTW in cancer patients experiencing decreased work ability after surgery. Therefore, physical therapists should actively promote health and employment support. In the future, the physical therapist will be required to acquire professional qualifications as a Coordinator of the Promotion of Health and Employment Support.

## Acknowledgments

We would like to thank the staff of the University of Occupational and Environmental Health for their cooperation in this study.

## Funding

This study was supported by the Workers’ Disease Clinical Research Projects Grant-in-Aid (230301-01) of the Ministry of Health, Labour and Welfare of Japan.

## Consent for Publication

In accordance with the Declaration of Helsinki and the Ethical Guidelines for Medical and Health Research Involving Human Subjects, the case report was explained to the subject, and written informed consent was obtained, taking into consideration the protection of personal information.

## Conflicts of Interest

The authors declare that they have no conflicts of interest.
